# The promising novel therapies for familial hypercholesterolemia

**DOI:** 10.1002/jcla.24552

**Published:** 2022-06-17

**Authors:** Ruoyu Chen, Shaoyi Lin, Xiaomin Chen

**Affiliations:** ^1^ School of Medicine of Ningbo University Ningbo China; ^2^ The Affiliated Ningbo First Hospital School of Medicine of Ningbo University Ningbo China; ^3^ Ningbo First Hospital Affiliated to School of Medicine of Zhejiang University Ningbo China

**Keywords:** familial hypercholesterolemia, gene therapy, lipidology, precision medicine

## Abstract

**Background:**

The incidence of premature atherosclerotic cardiovascular disease in familial hypercholesterolemia (FH) is high. In recent years, novel therapeutic modalities have shown significant lipid‐lowering ability. In this paper, we summarize the recent developments in novel therapies for FH via the treatment of different targets and discuss the characteristics of each targeted therapy. Based on the process of protein synthesis, we attempt to summarize the direct‐effect targets including protein, RNA, and DNA.

**Methods:**

For this systematic review, relevant studies are assessed by searching in several databases including PubMed, Web of Science, Scopus, and Google Scholar. The publications of original researches are considered for screening.

**Results:**

Most drugs are protein‐targeted such as molecule‐based and monoclonal antibodies, including statins, ezetimibe, alirocumab, evolocumab, and evinacumab. Both antisense oligonucleotide (ASO) and small interfering RNA (siRNA) approaches, such as mipomersen, vupanorsen, inclisiran, and ARO‐ANG3, are designed to reduce the number of mRNA transcripts and then degrade proteins. DNA‐targeted therapies such as adeno‐associated virus or CRISPR–Cas9 modification could be used to deliver or edit genes to address a genetic deficiency and improve the related phenotype.

**Conclusion:**

While the therapies based on different targets including protein, RNA, and DNA are on different stages of development, the mechanisms of these novel therapies may provide new ideas for precision medicine.

## Graphical abstract

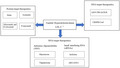

## 1 INTRODUCTION

Familial hypercholesterolemia (FH) is an autosomal codominant genetic disease characterized by high serum levels of low‐density lipoprotein cholesterol (LDL‐C).^1^ The disease is diagnosed by the presence of mutations in low‐density lipoprotein receptor (LDLR), apolipoprotein (ApoB), or proprotein convertase subtilisin/kexin 9 (PCSK9).^2^ The LDLR variant is the most common cause of FH and leads to the malfunction of the LDLR and a defect in the removal of LDL‐C from blood. Less common are pathogenic variants in the ApoB gene, which encodes apolipoprotein B100 (ApoB100), and these variants may be the predominant cause of 5%–15% of FH cases, leading to reduced binding of LDL‐C to LDLR. Gain‐of‐function (GOF) mutations in the PCSK9 gene account for 1% of reported FH cases, such mutations increase the destruction of LDLR on the hepatocyte surface and accordingly result in an increase in levels of circulating LDL‐C.^3^

The global morbidity rate of FH is between 1:200 and 1:300 with limited therapies.^4^ Patients with Heterozygous FH (HeFH) in China usually have a 3.5‐ to 16‐fold increased risk of coronary artery disease (CAD) in comparison with non‐FH patients. If untreated, FH would lead to premature atherosclerosis and an increased risk of cardiovascular events.^5^ The decrease in LDL‐C levels has been explicitly shown to mitigate cardiovascular risk.^6^, ^7^ The goal of the first‐line treatments is to limit cholesterol and saturated fats via a healthy way of living.^8^ However, lifestyle alone is insufficient for maintaining the normal average blood LDL‐C levels in patients with FH. Consequently, the use of lipid‐lowering therapy is vital. The pharmacological options now available include statins, bile acid sequestrants, fibrates, and cholesterol absorption inhibitors.^9^, ^10^ Although advances have been made in potential therapies and treatment of plasma lipids shows improvement, certain patients with FH fail to meet the treatment targets for LDL‐C.^11^ In addition, it is sometimes very difficult for traditional pharmacotherapy to be effective for patients with homozygous FH (HoFH), unless pharmacotherapy is coupled with LDL apheresis. Although LDL apheresis or orthotopic liver transplantation has been considered to be an optional therapeutic method for patients with HoFH, the complex and risky operation, severely limited availability of suitable donors, surgical risks, and perpetual use of immunosuppressive drugs after transplantation poses great challenges.^12^, ^13^

Currently, the majority of clinical therapies for patients with FH are small‐molecule‐ or antibody‐based approaches. It is important to develop new lipid‐lowering therapies with novel mechanisms of action and favorable side effect profiles.^14^ We assume that the expansion of novel FH therapy options may have the potential to bring about considerable economic and social benefits.

In this review, we propose novel therapies for FH according to the different targets—proteins, mRNAs, and DNA. In molecular biology, such therapies depend on the different stages of central dogma: the transcription of DNA into mRNA and the translation of mRNA into proteins.^15^

## 2 PROTEIN‐TARGETED THERAPEUTICS

In the clinic, the most commonly used drugs are small compounds. These drugs interact with target proteins to influence biological functions.

### 2.1 Statins

Protein‐targeted therapeutics such as statins can inhibit receptors or enzymes in the cytoplasm or on the cytomembrane. Statins are small‐molecule lipid‐lowering drugs that act on proteins in nanogram to microgram.^16^

Statins are hydroxymethylglutaryl coenzyme A (3‐hydroxy‐3‐methyl‐glutaryl‐CoA, HMG‐CoA) reductase inhibitors. In clinical practice, statins are the first‐line treatments for patients with FH in terms of pharmacotherapeutic management.^17^ Statins interfere with cholesterol biosynthesis by restricting the key step in the biosynthesis of isoprenoids and sterols, and this process is catalyzed by HMG‐CoA.^18^ In a randomized controlled trial (RCT) of patients with HeFH, daily administration of 80‐mg atorvastatin resulted, on average, in a 50% decrease in LDL‐C levels from baseline. In addition to reducing LDL‐C levels, statins also mitigate the risk of cardiovascular disease (CVD) and improve certain outcomes in prognosis.^7^, ^19^

Although the options for lipid‐lowering therapy have increased markedly since statins were introduced for clinical applications in 1987, only 10%–25% of diagnosed patients have received appropriate therapy, and many individuals have failed to achieve their LDL‐C goals.^20^ At present, most drugs interact with proteins, and they often bind to nontarget proteins or produce adverse effects via unnoticed interactions.^21^ Some patients are intolerant to statins and are easily affected by side effects such as myalgias and weakness.^22^Furthermore, statins upregulate the expression of not only LDLR but also PCSK9, which may limit the pharmacological effects of lowering LDL‐C levels. This is the “paradoxical effect” of statin management.^23^

### 2.2 Ezetimibe

Studies show that adding other lowering agents to the statins may further lower LDL‐C with few side effects. In the clinic, the Food and Drug Administration (FDA) approved the ezetimibe in 2002. Ezetimibe, a cholesterol absorption inhibitor, is used as an adjunct to statin for further reduce LDL‐C. It works by blocking the Niemann‐Pick C1‐Like (NPC1L1) receptor, which is a multipass membrane protein, expressed in the small intestine and liver. The NPC1L1 plays an important role in the process in cholesterol movement into the enterocyte.^24^ Individuals with the non‐GOF mutation of the NPC1L1 gene have lower levels of LDL‐C and a corresponding risk of ASCVD.^25^ In addition, the ezetimibe upregulates LDLR in the liver to reduce the circulating LDL‐C; it is an independent mechanism to statins.^26^ Many research explored the relationship between the additive effects of statins and ezetimibe. It provided an additional 23.4% reduction in LDL‐C compared with single statins therapy. What is more, the drug combination reduced high‐sensitivity C‐reactive protein (CRP).^27^

Nowadays, while there has undertreatment of FH, mainly treatment priorities for drugs are statins, ezetimibe, and bile acid bind resins.^28^

### 2.3 Alirocumab and evolocumab

Protein‐targeted therapeutics also include antibody‐based PCSK9 inhibitor drugs such as alirocumab and evolocumab, which block proteins in plasma at microgram to milligram.^29^ PCSK9 is an intrinsic protein that modulates the amount of LDLR and is very important in LDL‐C metabolism. Moreover, PCSK9 is secreted from hepatocytes into blood and binds to LDLR, which targets LDLR for lysosomal degradation.^30^ The loss‐of‐function (LOF) mutation of PCSK9, which was found to decrease LDL‐C levels, reinforces the fact that PCSK9 could be a target for therapy.^31^ The two antibodies currently in use against PCSK9 are fully human IgG subtypes that modify this pathway by binding to circulating PCSK9 and preventing it from binding LDLR.^32^ This process results in a lack of PCSK9 and leads to a large accumulation of LDLR on the membranes of liver cells. Ultimately, the removal of LDL particles is accelerated, and circulating LDL‐C levels are substantially reduced.^33^ Subcutaneous injection of PCSK9 inhibitors bound all newly secreted PCSK9 in the serum within hours of administration, and the effect lasted for the next few days.^34^, ^35^

However, this approach results in a large accumulation of the compound in the blood, with an average 10‐fold increase. For some patients, the total concentrations would be increased 20‐fold.^36^ As part of the immune complex, clearance of PCSK9 may be slow, and the return of circulating PCSK9 to the liver would be inhibited. This may stimulate counterregulatory transcriptional pathways that enhance PCSK9 synthesis and secretion.

### 2.4 Evinacumab

Traditional lipid‐lowering therapies, such as statins and PCSK9 inhibitors, work by upregulating LDLR expression, but there is virtually no activity in individuals with two null alleles, such as those with HoFH. Evinacumab is a monoclonal antibody against angiopoietin‐like protein 3 (ANGPTL3); it has received FDA approval in the United States and can confer a potential benefit in HoFH.^37^ Both ANGPLT3 LOF variants and pharmacologic inhibition of ANGPTL3 can lower LDL‐C levels without LDLR activity.^37^ ANGPTL3 is a secreted hepatic glycoprotein that disrupts the clearance of circulating LDL‐C by inhibiting lipases such as endothelial lipase and lipoprotein lipase, which hydrolyze triglycerides and phospholipids in circulating lipoproteins.^38^ Genome‐wide association and exome sequencing studies have found correlations between the LOF genetic variants of ANGPTL3 and a reduction in plasma LDL‐C, high‐density lipoprotein cholesterol (HDL‐C), and triglyceride levels for cardiovascular prevention.^39^, ^40^, ^41^ Prior to these findings, a combined hypolipidemia phenotype was observed in ANGPTL3 knockout mice.^42^ In a phase 3 trial, the LDL‐C level was decreased from baseline in the evinacumab group compared with the placebo group, which led to a 49.0% difference in LDL‐C levels and a 47% difference in triglyceride levels between the groups at 24 weeks, regardless of the degree of their LDLR function.^37^ Evinacumab was first approved in the USA on February 11, 2021, as supplementary therapy for LDL‐C reduction for adult and pediatric patients with HoFH who were older than 12 years, and it has received a positive response in the European Union.^38^

However, the use of antibody‐based drugs may create problems such as patient compliance, problems with the injection technique, and so on. For evinacumab, the main side effects include nasopharyngitis, influenza‐like illness, headache, and infusion‐site pruritus.^37^ Therefore, more options are needed because of the interactions and mechanisms of these drugs.

In summary, discrimination between the primary targets and off‐targets is necessary in the development of protein‐targeted drugs. The related phenotypes, including the curative effects and side effects, are dependent on the activation or inhibition of target proteins to a large extent.

## 3 RNA‐TARGETED THERAPEUTICS

Small molecules that affect proteins by inhibiting enzyme function or receptor activity have no therapeutic effect on proteins without enzyme activity. In addition, it may be difficult to achieve adequate concentrations of humanized monoclonal antibodies in circulation because of tolerance and cost considerations.^43^

**FIGURE 1:**
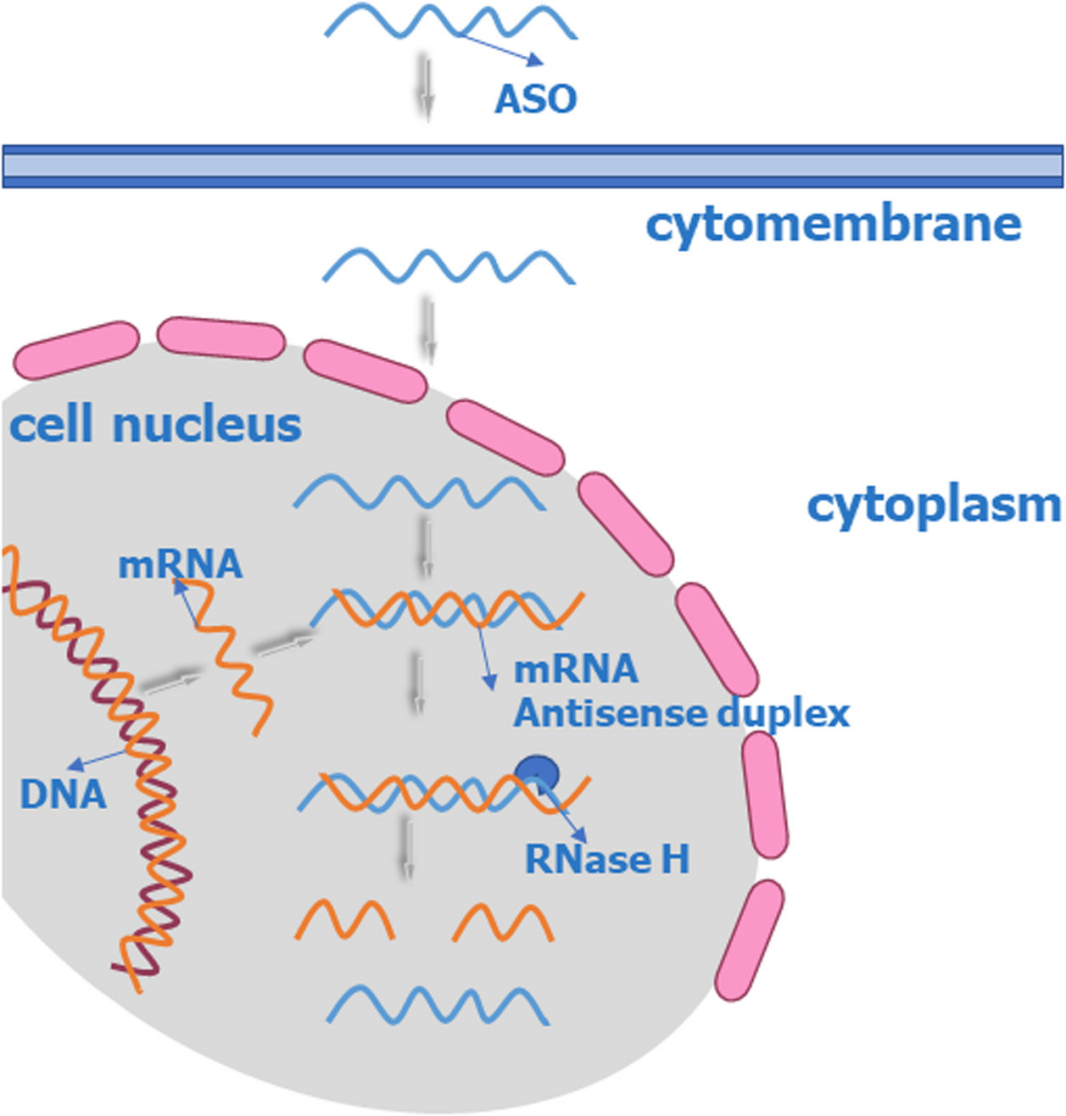
Antisense oligonucleotide‐based approaches: ASO utilizes a single‐stranded RNase H mechanism

**FIGURE 2:**
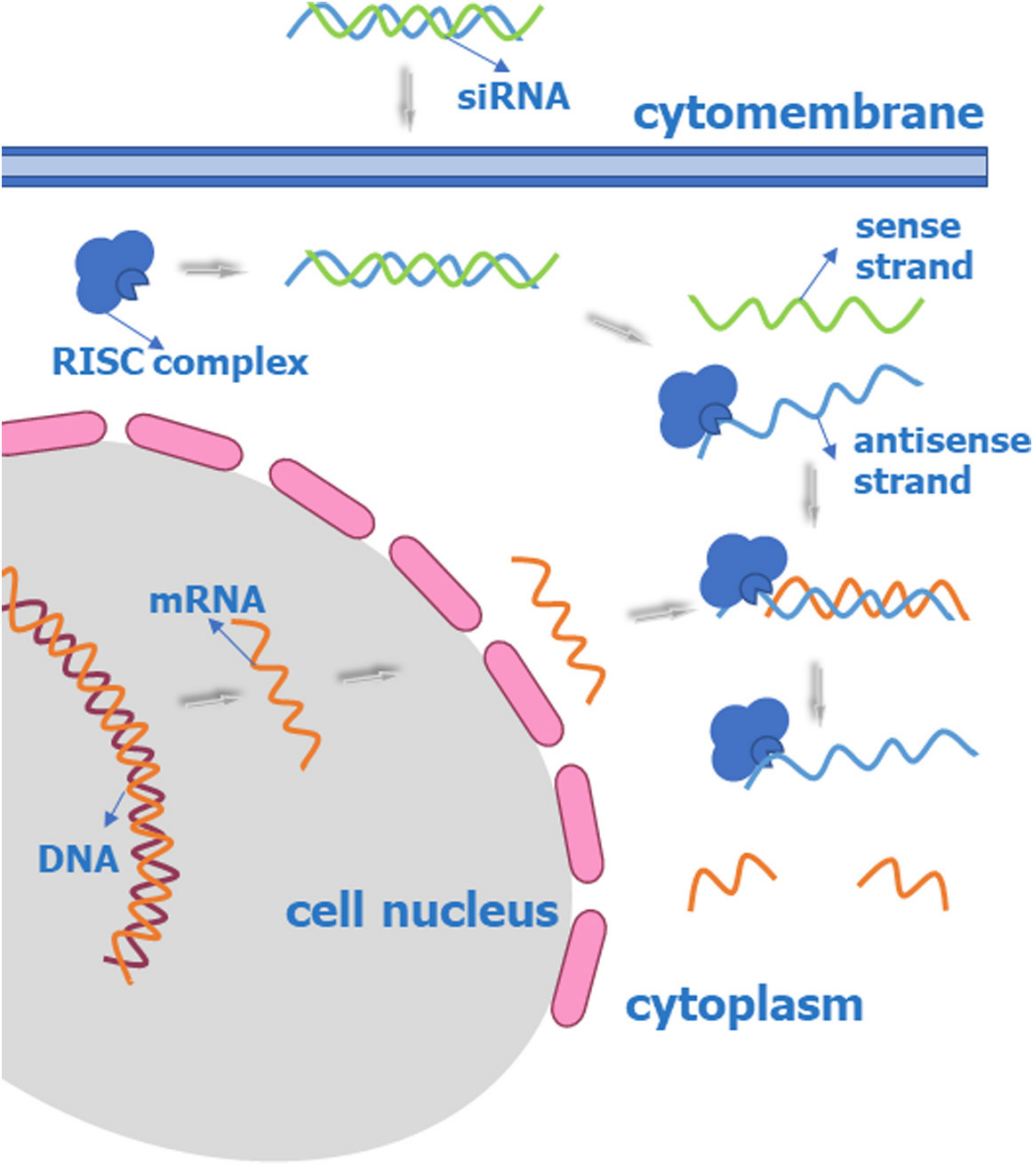
siRNA‐based approaches: siRNA technology employs a double‐stranded RISC mechanism

RNA‐targeted therapeutics seem to be a novel and elegant approach. It can act to regulate genes by directly targeting the nucleic acids that encode the proteins by interfering with the translation of mRNA into protein. Furthermore, RNA‐targeted therapeutics, which represent a drug discovery platform involving oligonucleotides, are akin to small‐molecule therapeutics and include small interfering RNA (siRNA), antisense oligonucleotide (ASO) (Figures 1 and 2), oligonucleotide‐induced alternative splicing, anti‐miRs, miRNA mimics, and mRNA upregulation.^44^ They are synthetic and small, do not integrate into the host genome, and have limited duration and activity.^45^

ASOs or siRNA can bind to the mRNA of disease‐causing genes and block their activation.^46^, ^47^ Notably, N‐acetylgalactosamine (GalNAc) modification of ASOs or siRNA by the asialoglycoprotein receptor could facilitate selective uptake into hepatocytes and ultimately significantly increase hepatic uptake.^48^ Therefore, these approaches could precisely and selectively match the specific mRNA in targeted organs or cells. In general, the new generation of RNA‐targeted drugs has enhanced potency, thereby reducing plasma drug levels, improving efficacy, and possibly reducing side effects.^49^

### 3.1 Antisense oligonucleotides (ASOs)

Since the 1970s, ASOs have frequently been used for silencing gene expression and can lower the levels of specific mRNAs in a safe and selective manner.^50^ ASOs are single‐stranded molecules composed of modified DNA. The middle bases are DNA, while those on either side are usually modified DNA that has been corrected, often containing 2′ methoxyethyl. An unspecified number of phosphorothioate moieties often replace the phosphodiester backbone, while the nucleic acid bases are not modified. The chemical modifications contribute to increased plasma stability and high affinity and binding to mRNA. This is the key reason that ASOs can be used as a drug, unlike unmodified native oligonucleotides, which degrade promptly in circulation.^43^ After subcutaneous injection, ASOs bind to circulating proteins, reach the liver, enter the cells, and then reach the nuclear membranes for efficacy. Because of their amphipathicity, ASOs can be dissolved in normal saline and be injected subcutaneously with no carrier. The basic premise of the use of ASOs is the ability to administer short‐ and single‐stranded nucleic acid sequences that bind to complementary RNA substrates. Then, the ASOs bind to specific sense mRNAs in cells within the nucleus or cytoplasm by Watson–Crick base pairing; finally, the resulting complex competitively inhibits translation or is degraded (Figure 1).^51^, ^52^ ASOs have rapid plasma uptake and a short distribution life (<1 h) but have a relatively long intracellular life (2–4 weeks), consequently having a long‐lasting effect. They are then cleaved to short fragments by exonucleases and endonucleases.^53^ Second‐generation ASOs are composed of sequence‐specific duplexes of the target mRNA. The duplexes serve as a substrate for RNase H, which is an endogenous mechanism in cells for cleaving ASO‐bound RNA, resulting in the degradation of target mRNA and ultimately in a decrease in the translation of the corresponding protein.^54^, ^55^

#### 3.1.1 ApoB100‐specific antisense oligonucleotides: Mipomersen

Mipomersen (Kynamro) is a second‐generation 20‐nucleotide ASO that binds to ApoB100 mRNA in the liver and creates a substrate for intrahepatic endonucleases. Then, the transcripts of ApoB100 are degraded, thereby reducing plasma LDL‐C concentrations.^56^ ApoB100 is one of two isoforms from the RNA transcript of the human ApoB gene, which has great importance in lipoprotein metabolism. And, ApoB100 is a vital structural component of very low‐density lipoprotein (VLDL) and LDL, which are synthesized by the human liver. In the early phase of atherosclerosis, dysfunctional arterial endothelial cells allow LDL to enter the endothelial layer and then adhere to intimal proteoglycans by way of ApoB, where it accumulates gradually in the intimal layer.^57^, ^58^ The genetic basis of non‐ApoB family hypobetalipoproteinemia (FHBL) suggests the possibility of ApoB as an attractive target for lipid‐lowering therapy.^59^ FHBL is an autosomal dominant disease characterized by low LDL‐C and ApoB levels, which may be due to the mutation of ApoB.^59^

Mipomersen includes 10 2′‐O‐methoxyethyl modifications, and there are five modifications at each end of the oligonucleotide that increase the affinity of target binding, prolong the half‐life in the target organ, and resist degradation by nucleases.^60^ In the center, the phosphorothioate nucleotides promote RNase H‐mediated cleavage.^55^ The plasma concentrations of mipomersen peak within 4 h after administration. Mipomersen is metabolized in tissues as a chain‐shortened metabolite, which is initially accomplished by endonucleases and produces oligonucleotides that have no further pharmacological effects. The smaller oligonucleotides are further digested by exonucleases and excreted mainly in the urine.^61^ Studies have shown that in healthy adults, mipomersen could increase the fractional catabolic rate of ApoB in both VLDL and LDL, thereby decreasing the levels of lipoproteins that contain ApoB.^62^ Phase I and II studies showed that mipomersen produced dose‐dependent decreases in all ApoB‐containing lipoproteins. In phase III trials, mipomersen was effective in patients with HoFH and patients with HeFH.^63^ In a primary phase III study, patients older than 12 years who were clinically diagnosed with or genetically confirmed to have HoFH received the maximum tolerated dose of a lipid‐lowering drug and were assigned to receive mipomersen 200 mg or placebo weekly.^64^ LDL‐C levels decreased by a mean percentage of 24.7% in all subjects after 6 months.^65^ In randomized trials and the open‐label extension phase, long‐term mipomersen treatment of FH reduced the levels of atherogenic lipoproteins and led to a decrease in cardiovascular events.^66^

However, mipomersen is approved only for HoFH, and the response varies widely among patients. Loss of ApoB is also associated with on‐target adverse effects such as liver steatosis, possibly because all ApoB‐containing lipoproteins are targeted and there may be no mechanism by which the liver clears excess fat. The use of mipomersen is complicated by its side effects. For instance, hepatotoxicity, injection‐related side effects, hepatic fat, and the high cost of the medication are also limiting factors.^67^

#### 3.1.2 ANGPTL3‐specific antisense oligonucleotides: Vupanorsen

ANGPTL3 has been assessed as a potential target for lowering the levels of LDL‐C and triglycerides for CVD prevention.^68^ Vupanorsen (AKCEA‐ANGPTL3‐LRx) is an N‐acetyl galactosamine‐conjugated (GalNAc3‐modified) ASO targeted to the liver that selectively inhibits the synthesis of the ANGPTL3 protein. GalNAc3‐modified ASOs can target the ASO to the asialoglycoprotein receptor on liver cells, and it has a treatment effect similar to that of unconjugated ASOs, although at a 20–30‐fold lower dose. Therefore, it could reduce systemic exposure risk and be administered at significantly lower doses and intervals.^69^ In a phase 2 study, 105 patients had fasting triglycerides >150 mg/dl (>1.7 mmol/L), type 2 diabetes, and hepatic steatosis at baseline. The patients received vupanorsen for 6 months by subcutaneous injection of 40 or 80 mg monthly or 20 mg weekly. The vupanorsen groups achieved a statistically significant dose‐dependent reduction in triglyceride levels (44%) compared with the placebo group, but no changes in liver fat or HbA1c were observed. In addition, the reductions in apolipoprotein C‐III (58%), residual cholesterol (38%), total cholesterol (19%), non‐HDL‐C (18%), HDL‐C (24%), apolipoprotein B (9%), and LDL‐C (7%) were also dose dependent, and the frequent side effects were generally mild and occurred at the injection site.^70^

Vupanorsen is also distinct from evinacumab, which is a monoclonal antibody targeted to ANGPTL3. Vupanorsen acts in liver cells, but evinacumab acts in plasma. Whether this would cause a difference in effect is still not clear. Evinacumab appears to be deficient regarding compliance, cost, and an inability to self‐administer. This is due to the high intravenous dosages (15–20 mg/kg) or subcutaneous injections required each week.^71^ An increasing number of epidemiologic, genetic, and genome‐wide association studies suggest that reducing plasma ANGPTL3 levels by inhibiting hepatic ANGPTL3 synthesis will benefit apolipoprotein B levels and improve the metabolic measurements related to dyslipidemia and atherosclerosis.^72^ There was no correlation of the effect with the concentration; rather, it is related only to complete deficiency.^73^ Patients who are homozygous for LOF mutations in the ANGPTL3 gene showed additional metabolic benefits, such as lower circulating free fatty acid levels and better insulin sensitivity. In addition, vupanorsen could reduce ApoC‐III levels in response to the reduction in ANGPTL3 levels. It is well known that triglyceride levels may be related, but vupanorsen reduces plasma triglycerides by targeting hepatic ANGPTL3 independent of apoC‐III.^74^

According to available studies, the adverse effects of vupanorsen are generally mild and include flu‐like reactions and adverse effects at the injection site.^70^ Further studies are needed to explore the efficacy and safety of different doses of vupanorsen to maximize target engagement in a population of individuals with elevated non‐HDL‐C and triglyceride levels receiving statin treatment. In conclusion, the reduction in triglyceride and atherogenic triglyceride‐rich lipoprotein levels by vupanorsen may suggest a novel strategy for decreasing the CV risk for patients with FH.

### 3.2 Small interfering RNA(siRNA) molecules

Small interfering RNA (siRNA) is small double‐stranded RNA molecules that are critical regulators of eukaryotic genome expression and function. siRNA post‐transcriptionally influences the mRNA of target genes, ultimately degrading them and preventing translation.^75^ They degrade target mRNA by binding with the RNA‐induced silencing complex (RISC), after which the antisense strand of siRNA is loaded into the RISC through Watson–Crick base pairing for sequence‐specific cleavage of the mRNA.^76^ The RISC has endonuclease activity to specific mRNA, and then after cleavage, the cellular exonucleases degrade the resulting mRNA fragments.^77^ The complex of the siRNA strand and RISC can then be reused to target other complementary mRNAs, thereby providing more transcripts for RNA silencing and maintaining a long duration of efficacy for an extended period of several months. (Figure 2) siRNA has activity in somatic and germline lineages of various eukaryotic species. Small noncoding RNA regulates various important stages of genome function. Some examples are chromatin, chromosomal segregation, transcription, RNA processing, RNA stability, and translation.^46^ These molecules are also involved in protecting the genome from invasive nucleic acids.^78^ In most mammalian cells, long double‐stranded RNA cannot be used to silence a specific gene because it provokes the interferon response to defend against viruses and then prompts the shutdown of protein synthesis. In contrast, siRNA can evade the interferon response in mammals and generate specific and efficient gene silencing.^79^

#### 3.2.1 Inclisiran (ALN‐PCSsc PCSK9)

As shown in previous studies, PCSK9 inhibitors, such as evolocumab and alirocumab, have become promising new therapeutic approaches for LDL‐C reduction. In recent years, siRNA has also been used to inhibit PCSK9.^80^

Inclisiran (ALN‐PCSsc PCSK9) is a long‐acting, synthetic siRNA molecule that targets mRNA of PCSK9 to counteract it and reduce the production of intracellular PCSK9, resulting in a durable and substantial reduction in LDL‐C levels.^81^, ^82^ The inclisiran molecule consists of a guide strand and passenger strand. The two RNA strands are complementary. Once inclisiran molecules are incorporated into hepatocytes, the guide strand binds the RISC and hybridizes with the complementary mRNA of PCSK9, causing degradation.^83^ Finally, translation of the PCSK9 protein would be limited, resulting in a reduction in serum LDL‐C levels.^84^ Notably, as previously mentioned, when degradation of the transcript is initiated, the complex is delivered to the liver where it remains active for a long period, interfering with more mRNA.^85^ In phase 2 and phase 3 trials, inclisiran was found to decrease LDL‐C levels by approximately 50% when administered subcutaneously every 6 months. Inclisiran was well tolerated, and there were no severe adverse events; however, injection‐site side effects were common compared to placebo (2.6% vs. 0.9% in the ORION‐10 trial and 4.7% vs. 0.5% in the ORION‐11 trial), although they were basically mild and did not last long.^82^ Interestingly, patients with diabetes showed no additional effects after treatment with PCSK9‐targeted siRNA‐driven strategies.^86^ In December 2020, inclisiran was first approved in the EU for use in adult patients with primary hypercholesterolemia, including HeFH and nonfamilial hypercholesterolemia or mixed dyslipidemia, by subcutaneous injection twice yearly as a dietary supplement. Inclisiran is intended to be used in combination with statins or with statins combined with other lipid‐lowering treatments in patients who cannot meet LDL‐C targets on the maximum tolerated statin doses. Inclisiran can be used separately or in conjunction with other approaches for patients who have statin intolerance or contraindications.^87^

#### 3.2.2 ARO‐ANG3 (ANGPTL3 mRNA)

Previous results indicate that ANGPTL3 is a notable new therapeutic target. In addition to monoclonal antibodies and ASOs, siRNA therapies targeting ANGPTL3 are also being developed.

The siRNA ANGPTL3 mRNA (ARO‐ANG3) is a GalNAc3‐conjugated siRNA that effectively and persistently inhibits the transcription of ANGPTL3 mRNA in liver cells. The phase I/II clinical study of ARO‐ANG3 (NCT03747224) is ongoing. In the control group, ARO‐ANG3 treatment for 16 weeks at doses of 100, 200, and 300 mg injected subcutaneously reduced circulating levels of ANGPTL3 by 96%, triglycerides by 72%, and LDL‐C by 50%.^88^ In the FH group, ARO‐ANG3 reduced ANGPTL3 levels by 62%–92% at week 16 in a dose‐dependent manner. LDL‐C levels were reduced by 23%–37%, and TG levels were reduced by 25%–43% at doses of 100, 200, and 300 mg injected subcutaneously. For non‐FH patients, ANGPTL3 levels were reduced by 85%, and LDL‐C levels were reduced by 28%, comparable to those patients with FH.

As of May 2020, no drug‐related side effects or discontinuations were found. Most adverse events were mild. The most common adverse events of ARO‐ANG3 treatment are respiratory tract infection (RTI) in 30% of participants and adverse events at the injection site in 13% of participants.^89^

In general, whether the aforementioned siRNA treatment method would be an effective approach for targeting ANGPTL3 needs further observation.

## 4 DNA‐TARGETED THERAPEUTICS

Although RNA‐targeted therapies are used selectively to silence and interfere with gene expression, DNA‐targeted therapy is usually intended to achieve the opposite effect. This technique involves introducing functional gene copies to restore the function of a mutated gene.

According to previous studies, certain DNA sequences of a gene have been observed to change lipoprotein levels, such as the LDLR variant, which is the most common cause of FH.^90^ Some kinds of variants are considered pathogenic, such as missense and nonsense variants, insertion and deletion mutations, splicing mutations, and large‐scale DNA copy number variation (CNV).^91^ LDLR mutations could influence various processes involved in LDL‐C uptake and metabolism and are the key driver of LDL‐C clearance without substitutes in vivo.^92^ Approximately 75% of human LDLR is expressed in the liver and determines LDL‐C removal from the bloodstream.^93^ Patients with HoFH have biallelic LOF mutations in LDLR, and orthotopic liver transplantation effectively resolves the HoFH phenotype; however, the adverse effects and risks of transplantation and long‐term immunosuppression are limitations.^94^ Consequently, patients with HoFH are considered ideal candidates for gene delivery and mutation correction.

Accumulating evidence supports the concept that overexpression of a corrected copy of a gene with an LOF mutation, such as LDLR, by retrovirus,^13^ adenovirus, or adeno‐associated virus (AAV) can effectively ameliorate the phenotype.^94^ The first clinical trial of gene replacement therapy was performed in five patients with HoFH and used a recombinant murine retroviral vector that contained LDLR cDNA to transfect the cells from resected liver tissue *ex vivo*, after which the genetically modified cells were injected into patients through a portal vein catheter.^13^ Although the process is safe and well tolerated, the challenges of the technique and invasiveness are present, and the inefficiency of the genetic recombination of retroviral vectors is the major barrier causing relatively low efficacy.^95^ Since then, in vivo preclinical research has started to explore efficient and safe vectors for delivering genetic cargo directly to liver cells.^96^ Various methods for gene delivery have been studied in different kinds of animal models of hypercholesterolemia with varying degrees of success.^97^

AAV vectors have low immunogenicity and no pathogenicity in humans, so AAV vectors could be suitable for gene delivery therapy. AAV is a branch of the Parvoviridae family, and it has different serotypes with different organ‐specific tropisms.^98^, ^99^ More than 90% of LDL‐C catabolism is mediated by LDLR in the liver. Thus, AAV serotype 8 (AAV8) has been developed, which is a highly hepatotropic vector. A recombinant liver tropic AAV8 vector named AAV8.TBG.hLDLR is under development. This vector carries a human wild‐type (WT) LDLR transgene under the control of a liver‐specific promoter called thyroxin‐binding globulin. Recombinant LDLR‐expressing AAV8 vectors have high efficiency in gene transfer and stable hepatocyte transduction for up to 180 days (compared to other tissues in which transduction is 1000‐fold higher), and they significantly improve lipid levels. In HeFH or HoFH humanized mouse animal models, AAV8 gene therapy leads significant reduction in total and LDL‐C with safety.^100^ Studies have shown the efficiency of AAV vector transduction in human hepatocytes is lower than that in murine hepatocytes.^101^ Nonetheless, in the treatment of hemophilia B, recombinant FIX‐expressing AAV8 induced significantly higher serum FIX levels in mice than in human patients. Nevertheless, despite a reconversion rate of only 1%–7% in patients with hemophilia compared to individuals with a normal level, this vector could show significant clinical efficacy that lasted more than 3 years.^102^ The case of AAV8 is related to its use in HoFH. This is because HoFH individuals carrying LDLR variants with residual LDLR activity greater than 2% (LDLR‐defective) have a much better prognosis than individuals with LDLR variants with residual LDLR activity <2% (LDLR‐negative).^103^ According to the preclinical studies in preparation for a phase 1 clinical trial, AAV8.TBG.hLDLR had good safety.^104^, ^105^ The phase I and II trials of AAV‐based liver‐directed AAV8.TBG.hLDLR aiming to assess the safety and efficacy of the treatment for HoFH are currently underway (NCT02651675; AAV8‐mediated LDLR Gene Replacement in Subjects With HoFH). Subjects in that study are followed for 5 years. The FDA has approved two AAV‐mediated therapies. Other research in this area will allow further treatment applications to be developed, and the results of the trials for HoFH will generate new ideas for treating FH.

Genome editing can also permanently repair existing genes instead of introducing a WT gene with gene replacement therapy.

Recently, treatment strategies involving the clustered‐regularly interspaced‐short‐palindromic‐repeat/CRISPR‐associated gene 9 (CRISPR–Cas9) system, an efficient gene‐editing complex, have begun to appear. The RNA‐guided nuclease cleaves the strands of DNA at the target site. Then, the CRISPR–Cas9 system provides engineered donor DNA for insertion into the target site when the cell's standard DNA repair mechanism tries to initiate DNA repair.^106^

CRISPR/Cas9 therapy has been explored for most genes associated with the regulation of lipid homeostasis and the genes targeted in RNA therapeutics. Although gene editing may not be poised to be adopted in clinical practice in the foreseeable future, achievements with this technique in animal models are notable.^107^, ^108^ The first HeFH mouse model created by in vivo somatic gene editing, which does not pass mutations to the next generation, used AAV8‐delivered CRISPR/Cas9 and disrupted the LDLR gene in mice within 6 weeks, resulting in severe hypercholesterolemia and atherosclerosis.^109^ Moreover, gene editing of PCSK9 by CRISPR–Cas9 is accomplished in animals through different delivery methods and has revealed a clinically meaningful reduction of >30% in LDL‐C levels. This finding seems to suggest that permanent silencing of disease‐causing genes has tremendous therapeutic potential.^110^, ^111^ A variant of CRISPR–Cas9 named base editing does not need the DNA double‐strand break. In LDLR (−/−) mice, this method is used to disrupt the ANGPTL3 gene, and the lipid levels of the mice were largely reduced without off‐target mutagenesis.^111^

In another trial using the CRISPR–Cas9 system, T cells from HoFH individuals were effectively reprogrammed, and then pluripotent stem cells were induced.^112^ The study showed that LDLRs are present in the cell membrane, providing a potential therapeutic approach for FH. Another study described an efficient approach for the simultaneous base editing and reprogramming of fibroblasts using a CRISPR–Cas9 adenine base.^113^ In this study, the approach generated gene‐edited human‐induced pluripotent stem cells isolated from the skin biopsies of patients carrying pathogenic point mutations and showed restoration of LDLR activity after gene modification. The approach is presented as being highly efficient while significantly reducing the time of the in vitro cell culture, thereby reducing the risk of in vitro changes. This may provide a solid basis for further experiments in vivo.

In recent studies, somatic cell gene editing has been performed in vivo by using the CRISPR/Cas9 system transduced via AAV to treat FH induced by the LDLR mutation using germline editing in mouse models.^114^ In this study, AAV‐CRISPR/Cas9 was used to modify the point mutation in hepatocytes and was delivered subcutaneously into LDLRE208X mice carrying an FH‐related gene mutation. Studies have shown that AAV‐CRISPR/Cas9‐mediated homologous directed repair of LDLR gene correction can partly salvage LDLR gene expression in vivo and efficiently improve atherosclerosis phenotypes such as cholesterol, triglyceride, and LDL‐C levels.^114^ Therefore, we can infer that the CRISPR–Cas9 system may play an important role in the recovery of LDLR mutations or defect in many other genes, including PCSK9, ApoB, and ANGPTL‐3, and would be a broadly useful therapeutic approach for FH.

## 5 CONCLUSIONS AND PERSPECTIVES

Despite significant advances in the treatment of FH, compound HeFH and HoFH remain serious genetic disorders that affect the human quality of life. For these patients, conventional treatment may not be very effective. Residual LDLR activity is critical for a therapeutic response, so restoration of LDLR activity or achievement of an obvious lipid‐lowering effect is a challenging task. Furthermore, novel therapies with mechanisms of action independent of this pathway are being explored. Gene‐based treatments offer great potential and possibilities to regulate key points in lipid metabolism with high specificity and efficiency. Novel therapies provide methods to address hitherto undruggable targets. While the long‐term safety and efficacy of such strategies still need more exploration, these novel platforms are in different phases of clinical development (Tables 1 and 2), which may trigger a paradigm shift in the treatment of patients with FH, especially those who suffer from the severe clinical symptoms of HoFH or are intolerant to conventional lipid‐lowering treatment.

**TABLE 1:** Novel and emerging platforms for FH

Classify (platform)	Name	Target	Mechanism of actin	Biochemical effect	Stage	Dose
*Protein*						
Enzyme and substrate	Statin	HMG‐CoA	HMG‐CoA competitive combination	Reduce LDL‐C	Approved	daily po
Enzyme and substrate	Ezetimibe	NPC1L1	NPC1L1 inhibition	Reduce LDL‐C and hs‐CRP combined statins	Approved	daily po
Antigen antibody	Alirocumab, evolocumab	PCSK9	Antigen antibody reaction	Reduce LDL‐C and Lp(a)	Approved	sc, once or twice monthly
Antigen antibody	Evinacumab	ANGPTL3 mAb	Antigen antibody reaction	Reduce LDL‐C and apolipoprotein B	Approved	15 mg/kg iv monthly
*RNA*						
ASO	Mipomersen (Kynamro)	APOB	Anti‐APOB antisense	Reduce LDL‐C	Approved	200 mg sc once weekly
ASO	Vupanorsen (ANGPTL3‐LRx)	ANGPTL3	Anti‐ ANGPTL3 antisense	Reduce TG, non‐HDL‐C	Phases II	40‐80 mg sc monthly
siRNA	Inclisiran	Anti‐PCSK9 siRNA	PCSK9 mRNA degradation	Reduces LDL‐C	Approved	300 mg sc twice yearly
siRNA	ARO‐ANG3	ANGPTL3	ANGPTL3 mRNA degradation	Reduces LDL‐C	phases I	On study
*DNA*						
AAV8	AAV8.TBG.hLDLR (RGX‐501) RegenXbio, RGX‐501	LDLR	LDLR gene therapy	Reduces LDL‐C	Phases I and II	Single iv infusion
CRISPR/Cas9	/	ANGPTL3/PCSK9	PCSK9 gene CRISPR/Cas9 editing	/	Preclinical	/

Abbreviations: iv, intravenous; po, per os; sc, subcutaneous.

**TABLE 2:** The selected and latest clinical trials of novel and emerging platforms for FH

Name	Clinical trials	Population	Duration	Treatment arms	Primary endpoint	Efficacy	Safety
Alirocumab	ODYSSEY HoFH (NCT03156621)^123^	HoFH subjects (*N* = 69)	12 weeks	150 mg every 2 weeks	Percent change in LDL‐C from baseline	LDL‐C (−35.6%)	Similar to placebo
Evolocumab	OSLER‐1 (NCT01439880)^124^	FH subjects (*N* = 1255)	1 year and additional 4 years	420 mg monthly	Percent change in LDL‐C from baseline	LDL‐C (−56%, 57%, 56%, 56%)	Similar to placebo, 5.7% discontinuation
Mipomersen (Kynamro)	NCT00607373^66^	HoFH subjects aged 12 years and older (*N* = 51)	26 weeks	200 mg weekly	Percent change in LDL‐C from baseline	LDL‐C (−24.7%)	Injection‐site reaction (76% vs 24% in placo)
Vupanorsen (ANGPTL3‐L_Rx_)	TRANSLATE–TIMI 70 (NCT04516291)^125^	Non‐HDL‐C ≥ 100 mg/dl and triglycerides 150 to 500 mg/dl on statin therapy (*N* = 51)	24 weeks	80 mg, 120 mg, or 160 mg every 4 weeks, or 60 mg, 80 mg, 120 mg, or 160 mg every 2 weeks	Percent change in non–HDL‐C from baseline	Non–HDL‐C 22.0% 60 mg 2 weekly, 27.7% 80 mg 2 weekly	Injection‐site reaction (33.3%), ALT or AST >3× ULN (44.4%)
Inclisiran	ORION‐10^82^	LDL‐C > 1.8 mmol/L (*N* = 1561)	540 days	284 mg on day 1, day 90, and every 6 months over a period of 540 days	Percent change in LDL‐C from baseline	LDL‐C (−52.3%)	Injection‐site adverse events (2.6% vs. 0.9% in placo)
Inclisiran	ORION‐11^82^	ASCVD or ASCVD risk equivalent (*N* = 1617)	540 days	284 mg on day 1, day 90, and every 6 months over a period of 540 days	Percent change in LDL‐C from baseline	LDL‐C (−49.9%)	Injection‐site adverse events (4.7% vs. 0.5% in placo)
ARO‐ANG3	NCT03747224^126^	Healthy and dyslipidemia individuals (*N* = 93)	16 weeks	100 mg, 200 mg, 300 mg	/	ANGPTL3 (−96%), TG (−72%), LDL‐C (−50%)	Similar to placebo

Monoclonal antibodies against PCSK9 are a landmark therapy for patients with FH. They are combined with other lipid‐lowering treatment approaches to lower LDL‐C levels, and less frequent dosing provides greater convenience. In addition to PCSK9 suppression based on antibodies, a variety of RNA‐focused approaches have been attempted. Gene‐targeted treatments specifically intervene at the transcriptional level, for instance, by impeding the production of proteins via siRNA and ASOs at the mRNA level. If the DNA sequence is clearly noted to affect the level of a lipoprotein that causes certain disorders, it may be a target for an ASO or siRNA, which will integrate with the mRNA of the specific gene. Small molecules are usually found in plasma and cells at similar concentrations, while antibodies are found only in plasma and other extracellular compartments. ASOs and siRNAs are designed to reach higher concentrations in liver cells than in plasma.^49^

One of the important advantages of ASOs in comparison with siRNA is the markedly higher affinity for the targeted molecule.^80^ ASOs presumably act in the nucleus, while the activity of siRNA is confined to the cytoplasm,^115^ which may decrease off‐target hybridization and side effects. However, due to the relatively short duration of exposure in most clinical trials, the long‐term effects of ASOs and siRNAs are basically unknown. Thus, more investigations are needed.

In contrast to protein‐targeted therapy, gene‐based therapy is sometimes not feasible. Early trials with ASOs targeting PCSK9 were terminated due to renal tubular toxicity.^116^ Another option is to increase the production of a specific protein by introducing specific coding sequences such as LDLR via gene editing.

CRISPR/Cas9 is a very versatile approach with some controversies. This technique produces double‐strand breaks, which are usually restored by nonhomologous end joining but are just as likely to be repaired by faulty repair strategies.^117^ Alternatively, the homology‐directed repair is accomplished by careful nucleotide sequence reconstruction with homologous chromosomal DNA or by exogenous single‐strand template DNA to introduce a specific incorporation sequence.^118^ In addition, it is vital to distinguish somatic gene editing and germline gene editing. In 2018, the birth of the world's first twin girls to have their germ cells edited caused shock and outrage around the world.^119^ Germline editing refers to making changes to the genome in the embryonic stage, which would be passed on to future generations. This approach may create troubling ethical issues.^117^

One alternative, exosomes, are intracellular vesicles that play a significant role in intercellular communication.^120^ Studies have gradually shown that exosomes may be promising treatment vectors, and they can effectively convey mRNA, miRNA, and plasmid DNA to specific cells. Intracellular vesicles are naturally nanosized and easy to use and have no cytotoxicity or immunogenicity compared with viruses.^121^ They could encapsulate abundant LDLR mRNA and express LDLR in donor liver cells. In the recipient cells, encapsulated mRNA is stabilized and functional. Moreover, the phenotypes of animal models may eventually be reversed using exosomes.^122^

In summary, it is plausible to postulate that whether gene‐based therapeutics inhibit or enhance the targeted gene, appropriate drug affinity, and resistance to nucleases are the key points that need to be addressed and may help improve the efficiency and frequency of medication, which requires further research and development in chemical therapy. This would be an important step toward the progress of precision medicine. While novel therapies have entered clinical use, problems such as high cost and the specific needs of various subpopulations may arise. For rare and devastating diseases, gene therapy may play a decisive therapeutic role. However, there are significant ethical challenges related to the abuse of gene‐modifying techniques. As an example, the use of CRISPR/Cas9 technology for the deliberate editing of human embryos caused much controversy. This reminds us that we should remain vigilant to ensure the smooth development of gene therapy in a scientific and ethical manner. To sum up, it indicates that further research on different therapeutics for different targets will be helpful in providing new ideas for the individual treatment of various diseases.

## CONFLICT OF INTEREST

We do not have any conflict of interest.

## Data Availability

Data sharing is not applicable to this article as no datasets were generated or analyzed during the current study.
